# Lattice Boltzmann Simulation of Mass Transfer Characteristics in Catalyst Layer of High-Temperature Proton Exchange Membrane Fuel Cells

**DOI:** 10.3390/membranes16010030

**Published:** 2026-01-04

**Authors:** Shengzheng Ji, Guogang Yang, Hao Wang

**Affiliations:** Marine Engineering College, Dalian Maritime University, Dalian 116026, China

**Keywords:** lattice Boltzmann method, HT-PEMFC, catalyst layer, phosphoric acid transport

## Abstract

As a critical component of high-temperature proton exchange membrane fuel cells (HT-PEMFCs), the catalytic layer (CL) significantly influences the overall performance of these systems. In this study, a pore-scale lattice Boltzmann (LB) model was established to simulate the multi-component mass transport in the HT-PEMFC catalyst layer. Based on the reconstruction of CL, the transport behavior of phosphoric acid was simulated. The effects of different carbon carrier diameters, porosity values, and Pt/C mass ratios on the transport of phosphoric acid in CL were studied. The distribution of phosphoric acid and air concentration, as well as the electrochemical surface area, was qualitatively and quantitatively analyzed. Finally, the optimal design parameters of CL structure were determined. The results show that, with increases in carbon carrier diameter, porosity, and Pt/C mass ratio, the distribution of phosphoric acid concentration shows an upward trend, and the distribution of air concentration shows a downward trend. The optimal ranges of carbon carrier diameter, porosity, and Pt/C mass ratio are 50–80 nm, 60–70%, and 40–50%, respectively. This study provides a new idea for further understanding the mass transport mechanism in the HT-PEMFC catalyst layer and provides effective suggestions for the optimization design of the HT-PEMFC catalyst layer structure.

## 1. Introduction

A high-temperature proton exchange membrane fuel cell (HT-PEMFC) is an energy conversion device that can directly convert chemical energy into electrical energy. Due to its high operating temperature, HT-PEMFC has the advantages of simple water management, fast reaction kinetics, and high fuel tolerance, which has attracted the attention of many researchers in recent years [1,2,3,4,5,6,7,8,9,10]. However, due to the evaporation of water in the proton exchange membrane at high temperatures, the ordinary Nafion^®^ membrane cannot meet the working requirements of HT-PEMFC [11]. At present, most HT-PEMFCs use acid-absorbing or hydrophobic polymer binders (such as PBI), and proton conduction is achieved by doping phosphoric acid. However, excessive phosphoric acid will occupy a large number of pore channels, hindering the transport of oxygen within the pores, slowing down the reaction kinetics, and seriously hindering the commercial application of HT-PEMFC. Therefore, it is necessary to optimize the pore structure of the catalyst layer to balance the distribution of phosphoric acid and gas diffusion, thereby enhancing the performance and durability of fuel cells.

Chevalier et al. [12] used the pore network model to study the role of the microporous layer in the redistribution of phosphoric acid in the HT-PEMFC gas diffusion electrode. The results showed that the presence of MPL promoted the redistribution of PA in CL and inhibited the leaching of PA into the channel. A CL crack structure that can retain acid under high current density conditions can be designed to avoid the loss caused by phosphoric acid migration. Søndergaard [13] studied the phosphoric acid loss caused by the evaporation mechanism and evaluated the durability of membrane electrode assemblies (MEAs). It was found that temperature and cumulative airflow volume had great influences on phosphoric acid loss. Mack et al. [14] used SEM, X-ray microtomography, and atomic force microscopy (AFM) to study the microstructure and macrostructure of HT-PEMFC electrodes. The results show that the macroscopic structure of the electrode has no significant effect on the performance of the steady-state battery. In contrast, the microstructure plays a crucial role in the formation of the three-phase boundary among the catalyst, phosphoric acid, and reactant gas, which determines the activity of the electrode and the performance of the battery. In addition, the polytetrafluoroethylene-rich surface in the CL layer delays the immersion of phosphoric acid in the electrode and hinders the start-up performance. In order to visualize the invasion of phosphoric acid, Bevilacqua et al. [15] injected liquid phosphoric acid into the gas diffusion layer (GDL) of HT-PEMFC. The three-dimensional image of GDL was obtained by X-ray computed tomography, and the equivalent pore network was generated by OpenPNM for simulation. They found that MPL can limit the entry of liquid phosphoric acid into carbon fiber substrates. For the samples containing MPL, the overall saturation at the breakthrough of phosphoric acid was significantly reduced. Xia et al. [16,17] established a numerical non-isothermal three-dimensional model to study the effects of thickness and porosity on flow uniformity, diffusion flux, and ohmic resistance. The results show that the flow uniformity and ohmic resistance increase with the increase in thickness and porosity. The effects of thickness and porosity on the diffusion flux are opposite. The diffusion flux decreases with the increase in GDL thickness, but increases with the increase in porosity. T. Sousa et al. [18,19,20,21] established a two-dimensional isothermal model to study the polarization curve of fuel cells and the influence of transmission characteristics on performance. They found that the mass transfer loss has a key impact on the model results. The above studies on HT-PEMFC cannot quantitatively analyze the transport mechanism inside the cell. However, the lattice Boltzmann method (LBM) has been gradually developed and improved in recent years, and it has been widely used in the study of fluid dynamic behavior in fuel cells, due to its simple algorithm and easy operation [22,23,24,25,26]. Duan et al. [27] used the multi-relaxation time lattice Boltzmann method (MRT-LBM) to study the transport behavior of phosphoric acid in the assembly film and gas diffusion electrode (GDE) process. The results show that increasing the exposed surface area of polytetrafluoroethylene in CL can inhibit the entry of phosphoric acid into CL. However, so far, most of the research on the HT-PEMFC catalyst layer has focused on the macroscopic scale; there are few reports on the application of LBM to the simulation of HT-PEMFC, and the research on the mass transport of HT-PEMFC lacks understanding and support at the pore scale.

Macro-scale simulation ignores the non-uniformity and multi-scale characteristics of the real pore structure in the HT-PEMFC catalytic layer, making it difficult to reveal the mass transfer mechanism of the catalyst layer at the pore scale. Based on the above research status, this study investigated the mass transport behavior of HT-PEMFC from the aspect of pore scale, which has the advantages of low computational cost and the ability to capture the heterogeneous structure of complex catalyst layers. The transfer characteristics of phosphoric acid in the catalyst layer of HT-PEMFC were analyzed by LBM. Based on the reconstruction of CL, the transport behavior of phosphoric acid was simulated. The effects of different carbon carrier diameters, porosity values, and Pt/C mass ratios on the transport of phosphoric acid in CL were studied. The distribution of phosphoric acid and air concentration, as well as the electrochemical surface area, was qualitatively and quantitatively analyzed, and the optimal design parameters of CL structure were obtained. It provides a theoretical reference for the optimal design of CL structure and is of great significance to improving the overall performance and life of the battery.

## 2. CL Microstructure Reconstruction

**Figure 1:**
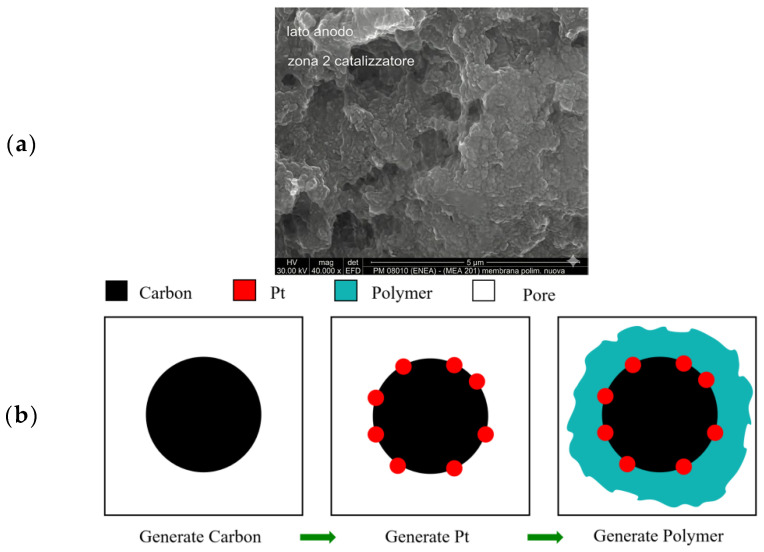
(**a**) SEM [29] images of the catalyst layer; (**b**) reconstruction process of the catalyst layer.

CL is a nanoporous structure composed of platinum particles dispersed on a randomly distributed carbon carrier. The size, surface morphology, content, and distribution characteristics of carbon carrier, platinum particles, and polymers are usually considered to be the most important factors affecting the transport of reactants in CL. Therefore, it is of great significance for the design and optimization of CL to clarify the real CL microstructure and understand its influence on reactant transport. At present, there are two methods to obtain the microstructure of CL. One is to prepare CL sample by experimental method, analyze the sample by CT imaging technology to obtain 2D structure image of CL, and, finally, use software to carry out 3D reconstruction [28]. Secondly, numerical reconstruction is carried out according to the microstructure characteristics of CL. Although a variety of advanced experimental techniques have been used to reconstruct the nanopore structure of CL, and can be used to obtain detailed structural information on the morphology, content, and distribution characteristics of related components, due to the complexity of its internal structure, obtaining high-resolution microstructure reconstruction still faces challenges. In addition, the experimental method of preparing CL is expensive, and it is difficult to ensure the consistency of the position of other components in the comparative study of different component distributions. At the same time, these experimental methods have low temporal and spatial resolutions, and it is not possible to observe the dynamic changes in the mass transfer process in real time. Compared with the experimental method, the numerical reconstruction method has the advantages of low cost and high efficiency, and can realize the optimization of different structural parameters. In order to investigate the phosphoric acid transfer characteristics within the catalyst layer, this study reconstructed the microstructure of the catalyst layer based on scanning electron microscopy (SEM) [29], as shown in Figure 1a. Figure 1b illustrates the reconstruction process, where the carbon support, platinum catalyst, and polymer electrolyte were sequentially added to the computational domain.

**Figure 2:**
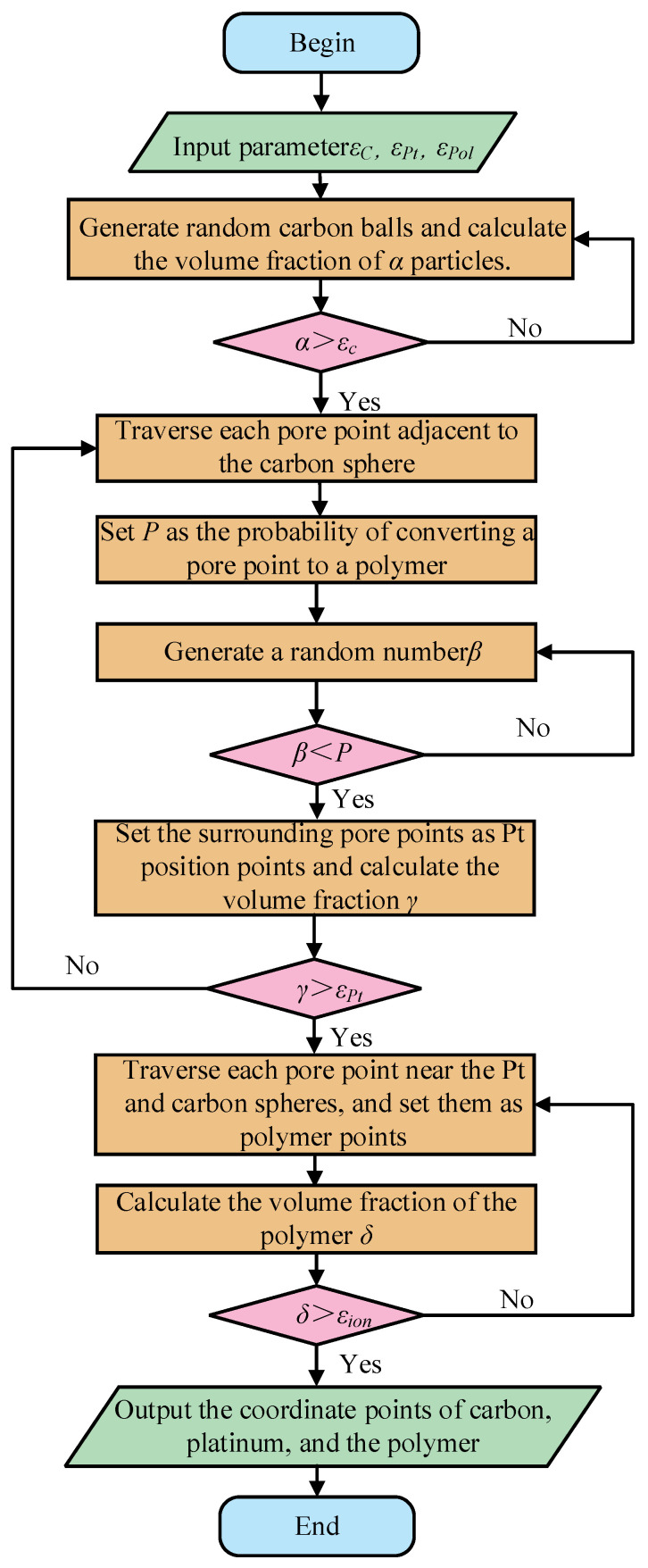
Flow chart of the CL reconstruction.

In this study, the two-dimensional microstructure of CL was reconstructed based on the method of Hou et al. [30], and the specific reconstruction process is shown in Figure 2. Before reconstruction, it was necessary to calculate the volume fraction of each material component; the expression is as follows:

(1) $$\varepsilon_{C}=(1-\varepsilon )\frac{1/\rho_{C}}{\theta_{Pt/C}/\rho_{Pt}+1/\rho_{C}+\left(1+\theta_{Pt/C}\right)\rho_{pol}/(\rho_{pol}(1-\theta_{pol}))}$$

(2) $$\varepsilon_{Pt}=(1-\varepsilon )\frac{\theta_{Pt/C}/\rho_{Pt}}{\theta_{Pt/C}/\rho_{Pt}+1/\rho_{C}+\left(1+\theta_{pt/C}\right)\rho_{pol}/(\rho_{pol}(1-\theta_{pol}))}$$

(3) $$\varepsilon_{pol}=(1-\varepsilon )\frac{\left(1+\theta_{Pt/C}\right)\rho_{ion}/(\rho_{pol}(1-\theta_{pol}))}{\theta_{Pt/C}/\rho_{Pt}+1/\rho_{C}+\left(1+\theta_{pt/C}\right)\rho_{pol}/(\rho_{pol}(1-\theta_{pol}))}$$

where $\varepsilon_{C}$, $\varepsilon_{Pt}$, and $\varepsilon_{pol}$ are the volume fractions of carbon, platinum, and polymer, respectively. ε is the porosity of CL. $\rho_{C}$, $\rho_{Pt}$, and $\rho_{pol}$ are the densities of carbon, platinum, and polymer, respectively. $\theta_{Pt/C}$ and $\theta_{pol}$ are the mass ratios of Pt/C and polymer, respectively.

In order to accurately display the microstructure of CL and take into account the limitation of computing power, the spatial resolution of a single lattice in this study was 4 nm, and the computational domain was 500 × 50 nm^2^. Assuming that the Pt particles had a grid with a side length of 1 lu, the microstructure of the carbon carrier was reconstructed with circular particles. The microstructure reconstruction parameters of CL in this study are shown in Table 1. The calculated values of ε, $\varepsilon_{C}$, $\varepsilon_{Pt}$, and $\varepsilon_{pol}$ were 0.39, 0.34, 0.023, and 0.24, respectively.

**Figure 3:**
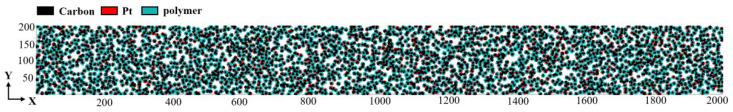
CL two-dimensional reconstruction structure.

According to previous studies on carbon carriers, the morphology of carbon carriers is often assumed to be a sphere [31,32,33,34,35]. In this study, a circle with a radius of 24–40 nm was randomly generated in the computational domain, and the volume fraction of the circle in the entire computational domain was calculated until the required volume fraction was reached. In addition, in order to ensure the uniformity of the distribution of the carbon carrier, the coincidence degree between the two adjacent carbon carriers cannot exceed 15%. According to the transmission electron microscope image, Pt particles were randomly distributed on the surface of carbon carrier. First, each pore point near the carbon carrier was traversed, and the probability that the pore point would be converted into polymer was set. The Pt position point was determined by comparing the value of the random number and the conversion probability until the target volume fraction of Pt was reached. In addition, in order to ensure the relative uniform distribution of platinum particles, the conversion probability should be as low as possible. For the reconstruction of the polymer, each pore point near the Pt and the carbon carrier was first traversed as the polymer position point, and then the integral number of the polymer object was calculated until the target volume fraction of the polymer was reached. It is worth noting that, in order to reduce the reconstruction error, the carbon carrier supports the Pt frame only once, when the Pt/C ratio is the same. The microstructure of CL generated according to the above method is shown in Figure 3, where black represents the carbon carrier, red represents Pt, turquoise represents the polymer, and white represents the pore. It can be seen from Figure 3 that all components of CL were evenly distributed, which met the expected goal. It is worth noting that the two-dimensional reconstruction model selected in this work mainly reconstructs the cross-section along the mass transfer direction, maintaining consistency with the typical HT-PEMFC catalytic layer in terms of porosity, volume fraction of each phase, and characteristic size. To a certain extent, it can represent the main diffusion path and local structural non-uniformity that control phosphoric acid transport.

## 3. Numerical Methods

### 3.1. MRT Pseudopotential Multiphase LB Model

The lattice Boltzmann method has been successfully used to address various two-phase transport problems [36,37,38,39]. In order to more clearly reveal the transport mechanism of phosphoric acid in the CL, the established model needs to have high accuracy and stability. Therefore, this study used a multiphase pseudopotential model with a multi-relaxation time (MRT) collision operator for simulation studies. The density distribution functions of different fluids are as follows:

(4) $$\begin{array}{l} f_{\sigma ,\alpha }(x+e_{\alpha }\Delta t,t+\Delta t)-f_{\sigma ,\alpha }\left(x,t\right)=-M^{-1}SM(f_{\sigma }(x,t)-f_{\sigma }^{eq}(x,t))\\ +M^{-1}\left(I-\frac{S}{2}\right){\bar{F}}_{\sigma } \end{array}$$

where *x* and *t* represent the lattice position and time, respectively. *σ* and *α* represent the direction of different components and the direction of discrete velocity, respectively. The right side of the equation describes the spatial and temporal changes in the time distribution function *f* to Δt. *I* is the identity matrix, and ${\bar{F}}_{\sigma }$ represents the external force term in the moment space. In this study, the D2Q9 model was used to solve the problem, where *M* and *S* represent the orthogonal transformation matrix and the diagonal relaxation matrix, respectively, as follows:

(5) $$M=\left[\begin{array}{ccccccccc} 1 & 1 & 1 & 1 & 1 & 1 & 1 & 1 & 1\\ -4 & -1 & -1 & -1 & -1 & 2 & 2 & 2 & 2\\ 4 & -2 & -2 & -2 & -2 & 1 & 1 & 1 & 1\\ 0 & 1 & 0 & -1 & 0 & 1 & -1 & -1 & 1\\ 0 & -2 & 0 & 2 & 0 & 1 & -1 & -1 & 1\\ 0 & 0 & 1 & 0 & -1 & 1 & 1 & -1 & -1\\ 0 & 0 & -2 & 0 & 2 & 1 & 1 & -1 & -1\\ 0 & 1 & -1 & 1 & -1 & 0 & 0 & 0 & 0\\ 0 & 0 & 0 & 0 & 0 & 1 & -1 & 1 & -1 \end{array}\right]$$

(6) $$S=diag(\tau_{\rho }^{-1},\tau_{e}^{-1},\tau_{\varsigma }^{-1},\tau_{j}^{-1},\tau_{q}^{-1},\tau_{j}^{-1},\tau_{q}^{-1},\tau_{\nu }^{-1},\tau_{\nu }^{-1})$$

Using the orthogonal matrix *M*, the distribution function *f* and the equilibrium distribution function $f^{eq}$ were transformed from velocity space to matrix space [40,41], as follows:

(7) m=M·f

(8) $$m^{eq}=M\cdot f^{eq}$$

(9) $$\begin{array}{c} m^{eq}=\rho {\left(1,-2+3{\left|v\right|}^{2},1-3{\left|v\right|}^{2},v_{x},-v_{x},v_{y},-v_{y},v_{x}^{2}-v_{y}^{2},v_{x}v_{y}\right)}^{T} \end{array}$$

where ρ is the macroscopic density of fluid, *v* is the macroscopic velocity, and the calculation equation is as follows:

(10) $$\rho_{\sigma }=\sum_{\alpha }f_{\sigma ,\alpha }$$

(11) $$v=\frac{\sum_{\sigma }\left(\sum_{\alpha }f_{\sigma ,\alpha }c_{\alpha }+\frac{F_{\sigma }}{2}\delta_{\iota }\right)}{\sum_{\sigma }\rho_{\sigma }}$$

where $F_{\sigma }$ is the force between fluids, which is divided into flow–flow and flow–solid forces. The equation is as follows:

(12) $$\begin{array}{l} F_{\sigma ,\mathrm{int}}(x)=-G_{\sigma \sigma }\psi_{\sigma }(x)\sum_{\alpha }w({\left|c_{\alpha }\right|}^{2})\psi_{\sigma }(x+c_{\alpha }\Delta t)c_{\alpha }\\ -G_{\sigma \bar{\sigma }}\psi_{\sigma }(x)\sum_{\alpha }w({\left|c_{\alpha }\right|}^{2})\psi_{\bar{\sigma }}(x+c_{\alpha }\Delta t)c_{\alpha } \end{array}$$

(13) $$F_{\sigma ,ads}(x)=-G_{\sigma w}\psi_{\sigma }(x)\sum_{\alpha }\nu ({\left|c_{\alpha }\right|}^{2})\psi_{\sigma }(x)s(x+c_{\alpha }\Delta t)c_{\alpha }$$

where $G_{\sigma \sigma }$ is the force parameter between the same fluid, $G_{\sigma \bar{\sigma }}$ is the force parameter between different fluids, and $G_{\sigma w}$ is the force parameter between fluid and solid. A larger gas–liquid density ratio was achieved by changing the expression of the potential function ψ of different components.

(14) $$\psi_{\sigma }=\left\{\begin{array}{cc} \sqrt{2(\kappa p_{EOS}-{\rho_{1}}^{2}C_{s})/G_{11}} & ,\sigma =1\\ \rho_{2} & ,\sigma =2 \end{array}\right.$$

where κ is used to adjust the thickness of the gas–liquid interface, which is related to the stability of the model. $p_{EOS}$ is the pressure of the phosphoric acid component, which was obtained by the Peng–Robinson equation.

Equation (4) is converted to Equation (15) through the spatial matrix.

(15) $$m^{\prime }=m-S(m-m^{eq})+\Delta t(I-\frac{S}{2}){\bar{F}}_{\sigma }+\Delta tC$$

where *I* is the identity matrix, *S* and *C* are the source terms, and the expression is as follows [42]:

(16) $${\bar{F}}_{\sigma }=\left[\begin{array}{l} 0\\ 6(v_{x}F_{x}+v_{y}F_{y})+\frac{12\sigma {\left|F\right|}^{2}}{\psi^{2}\delta_{t}(\tau_{e}-0.5)}\\ -6(v_{x}F_{x}+v_{y}F_{y})-\frac{12\sigma {\left|F\right|}^{2}}{\psi^{2}\delta_{t}(\tau_{e}-0.5)}\\ F_{x}\\ -F_{x}\\ F_{y}\\ -F_{y}\\ 2(v_{x}F_{x}-v_{y}F_{y})\\ \left(v_{x}F_{x}+v_{y}F_{y}\right) \end{array}\right],C=\left[\begin{array}{l} 0\\ 1.5\tau_{e}^{-1}(Q_{xx}+Q_{yy})\\ -1.5\tau_{e\zeta }^{-1}(Q_{xx}+Q_{yy})\\ 0\\ 0\\ 0\\ 0\\ -\tau_{v}^{-1}(Q_{xx}-Q_{yy})\\ -\tau_{v}^{-1}Q_{xy} \end{array}\right]$$

where σ is used to adjust the thermodynamic consistency.

(17) $$Q=\gamma \frac{G_{11}}{2}\psi (x,t)[\sum_{\alpha =1}^{8}w({\left|e_{\alpha }\right|}^{2})[\psi_{1}(x+e_{\alpha },t)-\psi_{1}(x,t)]e_{\alpha }e_{\alpha }]$$

where γ is used for surface tension adjustment. Finally, the matrix space was transformed into a velocity space, and a new distribution function was obtained, as follows:

(18) $$f^{\prime }=M^{-1}m^{\prime }$$

### 3.2. Computational Domain and Boundary Conditions

**Figure 4:**
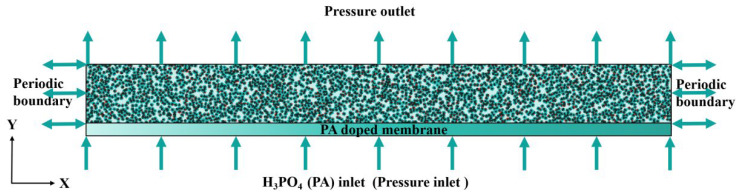
Schematic of computation domain.

The setting of boundary conditions is crucial for numerical simulations. In this study, to more accurately simulate the phosphoric acid transfer process, it is assumed that, at the initial moment, the proton exchange membrane is filled with phosphoric acid, the CL pores are filled with air, and phosphoric acid flows upward along the *Y*-axis. Both the inlet and outlet adopt the Zou–He pressure boundary conditions. Phosphoric acid is driven by a pressure difference to infiltrate the CL, while periodic boundary conditions are applied in all other directions. The internal obstacles in the CL use a nonslip rebound boundary. Considering the limitation of computing resources, the calculation area was set to 2000 × 200 lu^2^, as shown in Figure 4.

### 3.3. Model Validation

To accurately simulate the transport process of phosphoric acid in the CL, the established model needed to satisfy thermodynamic consistency for the fluid, large density ratio and viscosity ratio, independent regulation of surface tension, and validation through Laplace verification and static contact angle tests.

(1) Verification of thermodynamic consistency

**Figure 5:**
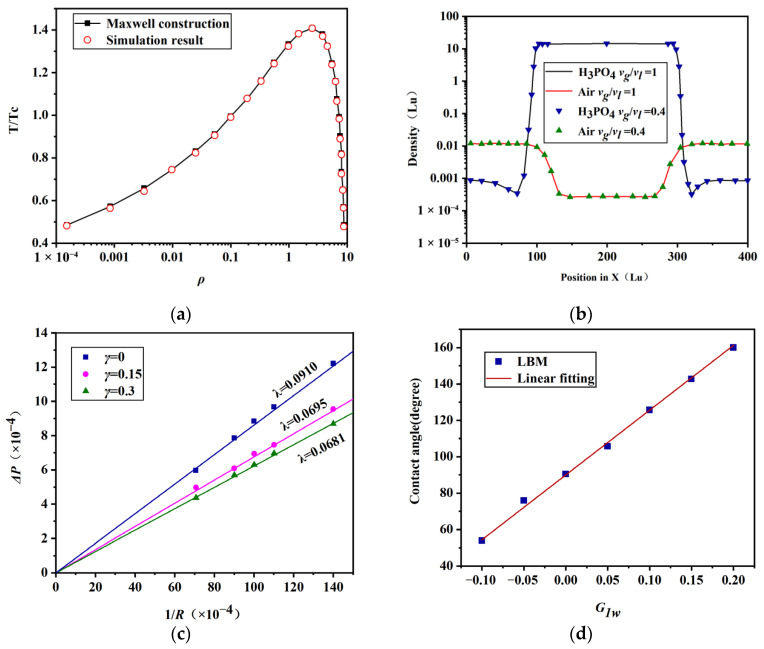
Model validation: (**a**) verification of thermodynamic consistency, (**b**) verification of large density ratio and viscosity ratio, (**c**) verification of surface tension-independent regulation and Laplace’s law, and (**d**) static contact angle test.

The Peng–Robinson equation was used to construct the Maxwell theoretical solution curve, and the MRT-LBM simulation results were compared with the theoretical solutions, validating the thermodynamic consistency of the pseudopotential MRT-LBM model used in this study. In the verification process, a stationary phosphoric acid droplet with a radius of 50 was placed in the center of the 400 × 400 calculation domain, and periodic boundary conditions were adopted in all surrounding directions. The initial density distribution in the calculation domain was determined by the following formula [43,44]:

(19) $$\rho (x,y)=\frac{\rho_{l}+\rho_{g}}{2}-\frac{\rho_{l}-\rho_{g}}{2}\tanh[\frac{2(r-r_{0})}{W}]$$

where $\rho_{l}$ is the density of liquid phosphoric acid, $\rho_{g}$ is the density of air, *r*_0_ is the radius of the circular droplet, and *r* is the distance between the coordinate point and the center of the circle. *W* is the width of the density transition interface. The simulation results are shown in Figure 5a. It can be seen that the simulation results are in good agreement with the analytical solution, and the LB model used in this study can ensure thermodynamic consistency at 0.46 Tc (25 °C).

(2) Verification of large density ratio and viscosity ratio

The density ratio and viscosity ratio are the key factors to simulate the dynamic behavior of phosphoric acid droplets. When the temperature is 25 °C, the density ratio of liquid phosphoric acid to air is close to 1500, and the kinematic viscosity ratio of air to liquid phosphoric acid is about 0.4. To achieve the realistic gas–liquid kinematic viscosity ratio, this study adjusted the value of $\tau_{\nu }^{-1}$. In the simulation verification, the gas–liquid kinematic viscosity ratios were set to 1 and 0.4, respectively, and the density distribution of phosphoric acid and air components was simulated. The simulation results are shown in Figure 5b. The results show that the adjustment of viscosity ratio does not affect the density distribution of the model, and the model can realize the real gas–liquid density ratio and viscosity ratio.

(3) Verification of surface tension independent regulation and Laplace’s law

The surface tension of the droplet can be calculated by Laplace ‘s law. According to Laplace ‘s law, when the droplet reaches a relatively stable state, the internal and external pressure difference in the droplet is proportional to the surface tension (λ) of the fluid and inversely proportional to the radius of curvature (*R*) of the droplet. The expression is as follows:

(20) $$\Delta p=\frac{\lambda }{R}$$

In this study, the computational domain grid was set to 100 × 100 lu^2^. At the initial moment, the droplet with a radius of *R* was placed at the center of the computational domain, and the densities of the two phases inside and outside the droplet were set to $\rho_{1}=3\times 10^{-3},\rho_{2}=1$. The surface tension could be adjusted by changing the parameter *γ* in Equation (17). The simulation results are shown in Figure 5c. The results show that, when the *γ* values are 0, 0.15, and 0.3, the corresponding surface tension lattice units are 0.0910, 0.0695, and 0.0681, respectively, and the relationship with 1/*R* is linearly correlated. The simulation results are in good agreement with Laplace’s law, which proves the accuracy of the model.

(4) Static contact angle test

By adjusting the interaction strength (*G_w_*) between the fluid and solid, the solid surface can exhibit different wettability. In the contact angle test, the computational domain grid was set to 100 × 100 lu^2^. At the initial time step, a hemispherical droplet of phosphoric acid with a radius *R* was placed at the center of the wall at Y = 0. By adjusting the interaction strength (*G_w_*) between the fluid and solid, the droplet eventually reached an equilibrium state. The simulation results are shown in Figure 5d. The contact angle changes almost linearly with the value of *G_1w_*, which indicates that the model can simulate different contact angles and can accurately deal with the interaction between solid and liquid.

## 4. Results and Discussion

### 4.1. Effect of Carbon Carrier Diameter on the Flow Behavior of Phosphoric Acid

**Figure 6:**
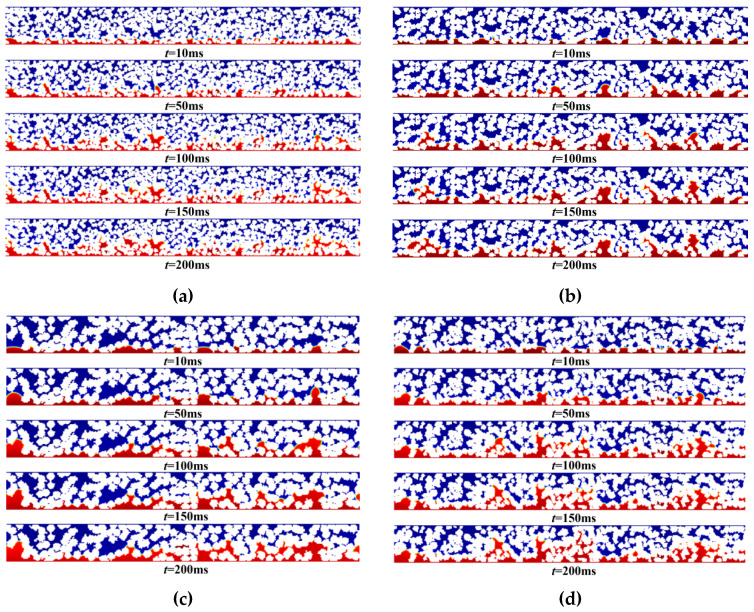
Phosphoric acid distribution at different carbon carrier diameter ranges: (**a**) *D*c = 30–50 nm, (**b**) *D*c = 50–80 nm, (**c**) *D*c = 80–100 nm, and (**d**) *D*c = 30–100 nm.

To study the effect of the carbon carrier diameter in the CL on phosphoric acid transport, this research selected carbon carrier diameters of 30–50 nm, 50–80 nm, 80–100 nm, and 30–100 nm, while ensuring the same porosity and Pt/C mass ratio. Figure 6a–d describe the phosphoric acid distributions when the diameters of the carbon carriers are 30–50 nm, 50–80 nm, 80–100 nm, and 30–100 nm respectively. It can be seen from Figure 6 that, as the diameter range of the carbon carrier increases, the diffusion area of phosphoric acid also expands. When the diameter range of the carbon carrier is 50–80 nm, the saturation of phosphoric acid within CL is relatively high. At the same time, as the diameter range of the carbon carrier increases, the diffusion degree of phosphoric acid gradually rises. This is because, with the increase in the carbon carrier diameter range, the porosity and connectivity of the CL increase, effectively reducing the tortuosity of the diffusion path, thereby making the pore structure more open and the pore distribution more uniform.

**Figure 7:**
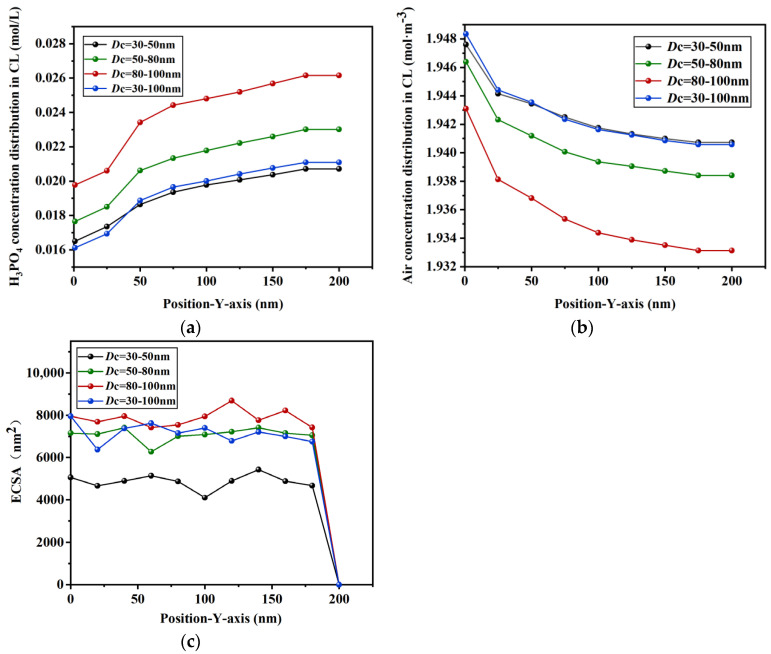
Concentration distribution and the electrochemical surface area along the *Y*-axis for different carbon carrier diameters: (**a**) H_3_PO_4_, (**b**) air, and (**c**) ECSA.

The concentration distribution of phosphoric acid and air along the *y*-axis, as well as the electrochemical surface area, were quantitatively calculated using MATLABR2003a for different carbon carrier diameters, and the results are shown in Figure 7. The results show that the phosphate concentration increases with the increase in the *Y*-axis distance, while the air concentration decreases with the increase in the *Y*-axis distance. At the same position, the phosphate concentration and electrochemical surface area increase with the increase in the carbon carrier diameter, while the air concentration decreases with the increase in the carbon carrier diameter. Phosphoric acid serves to transfer protons in HT-PEMFCs. A suitable amount of phosphoric acid infiltrating the CL is beneficial for the reaction process, while excessive phosphoric acid can lead to ‘acid flooding’ of the Pt catalyst, thereby reducing the reaction rate. Therefore, it is necessary to control the amount of phosphoric acid infiltration during the electrode assembly process. Through the above analysis of phosphoric acid concentration, it was concluded that a carbon carrier diameter in the range of 50–80 nm is more conducive to phosphoric acid transmission.

### 4.2. Effect of Porosity on the Flow Behavior of Phosphoric Acid

**Figure 8:**
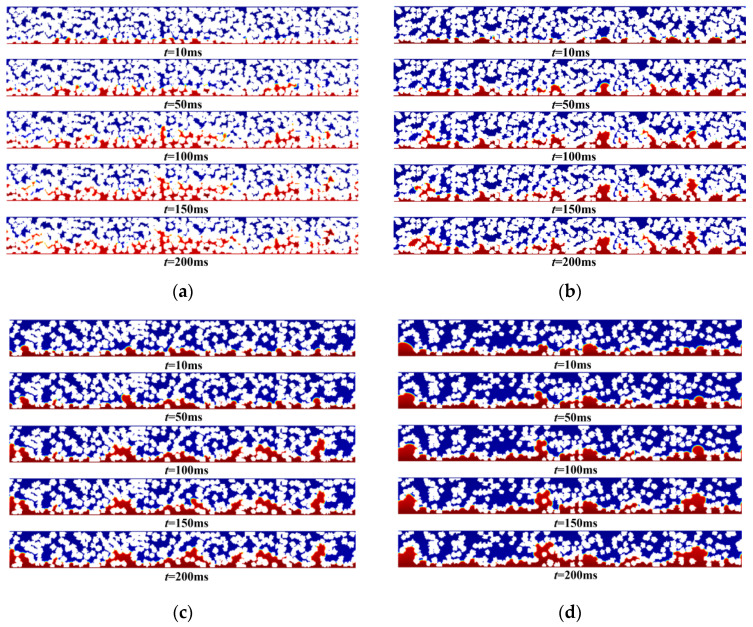
Phosphoric acid distribution at different porosities: (**a**) 40%, (**b**) 50%, (**c**) 60%, and (**d**) 70%.

Porosity is a key factor affecting the performance of HT-PEMFCs. A higher porosity helps to reduce mass transport resistance; however, excessively high porosity can lead to decreased mechanical strength of the CL structure and increased ohmic losses. Therefore, it is necessary to find a balance between porosity and mechanical strength to ensure the stability and enhance the performance of HT-PEMFC. In this study, CL microstructure models with porosity values of 40%, 50%, 60%, and 70% were established under the premise that the diameter of carbon carrier and the mass ratio of Pt/C remained unchanged, and the effect of porosity on the flow behavior of phosphoric acid was studied. The results are shown in Figure 8a–d. As can be seen from Figure 8a–d, with the increase in CL porosity, the phosphoric acid diffusion capacity increases, and the phosphoric acid distribution becomes more uniform. Within the same period of time, the higher the porosity, the higher the phosphoric acid saturation. This is because, as the porosity increases, the number of pores grows, the connectivity of pore channels is higher, and the pore distribution becomes more uniform. As a result, the resistance encountered during the diffusion of phosphoric acid is smaller, thereby enhancing the diffusion capacity of phosphoric acid.

**Figure 9:**
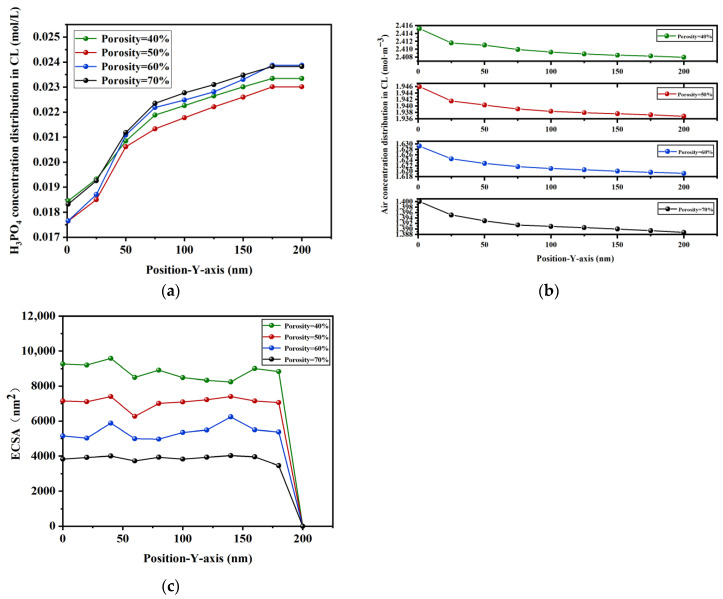
Concentration distribution and the electrochemical surface area along the *Y*-axis for different porosities: (**a**) H_3_PO_4_, (**b**) air, and (**c**) ECSA.

In addition, this study quantitatively calculated the concentration distribution of phosphoric acid and air along the *Y*-axis, as well as the electrochemical surface area, for different porosities, and the results are shown in Figure 9. The results show that the concentration of phosphoric acid increases with the increase in *Y*-axis distance, while the concentration of air decreases with the increase in *Y*-axis distance. At the same position, the greater the porosity, the greater the diffusion of phosphoric acid, while electrochemical surface area becomes smaller. Through comprehensive analysis, it was found that, when the porosity range is between 60% and 70%, the phosphoric acid transport efficiency is relatively high, which is more conducive to phosphoric acid transport.

### 4.3. Effect of Pt/C Mass Ratio on the Flow Behavior of Phosphoric Acid

**Figure 10:**
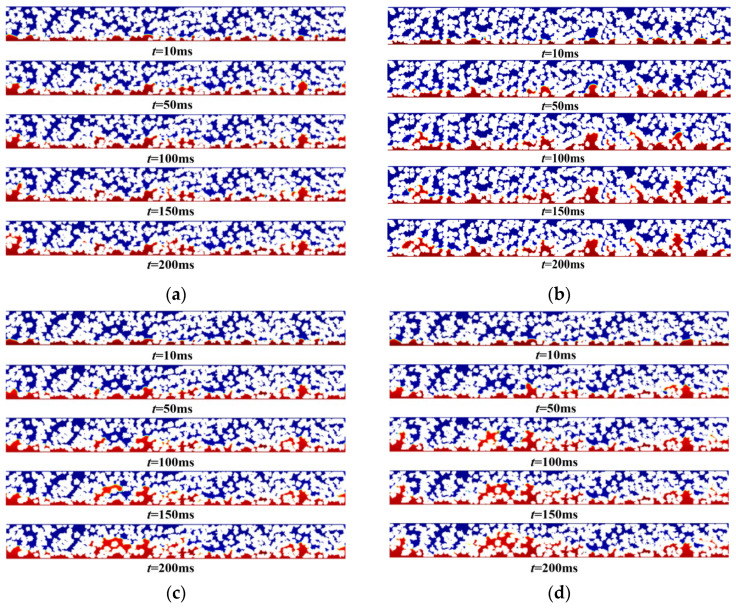
Phosphoric acid distribution at different Pt/C mass ratios: (**a**) 40%, (**b**) 50%, (**c**) 60%, and (**d**) 70%.

The Pt/C mass ratio is a key parameter for optimizing fuel cell electrode performance, significantly affecting the catalyst layer thickness and the density of active Pt sites. Although a higher Pt content can increase the densities of active sites, it also leads to an increase in catalyst layer thickness, which can cause mass transport limitations and potentially reduce fuel cell performance. Furthermore, excessive use of Pt not only wastes resources, but also significantly increases costs. Therefore, a reasonable Pt/C ratio design is crucial for balancing electrode kinetics and mass transfer performance, improving system efficiency and reducing economic costs. Based on the above research, the effect of the Pt/C mass ratio on the flow behavior of phosphoric acid was studied. The carbon carrier diameter was set to 50–80 nm, the porosity to 50%, and the Pt/C ratios to 40%, 50%, 60%, and 70%. The phosphoric acid distribution at different Pt/C mass ratios is shown in Figure 10a–d. As can be seen from Figure 10, with the increase in the Pt/C mass ratio, the phosphoric acid diffusion capacity gradually increases, and the phosphoric acid distribution is relatively uniform, but the degree of phosphoric acid diffusion varies little. This indicates that the influence of the Pt/C mass ratio on phosphoric acid diffusion is relatively small.

**Figure 11:**
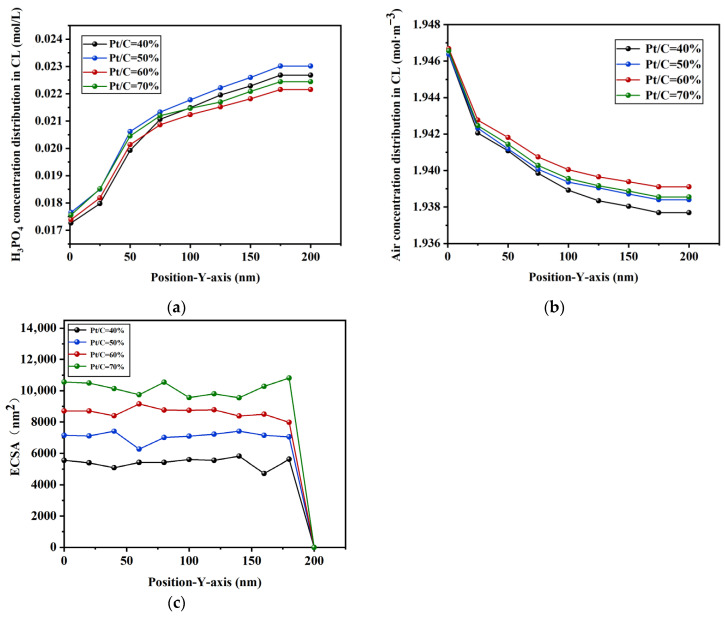
Concentration distribution and the electrochemical surface area along the *Y*-axis for different Pt/C mass ratios: (**a**) H_3_PO_4_, (**b**) air, and (**c**) ECSA.

In order to further investigate the effect of the Pt/C ratio on the flow behavior of phosphoric acid, a quantitative analysis was also conducted on the concentration distributions of phosphoric acid and air along the *Y*-axis, as well as on the electrochemical surface area at different Pt/C ratios. The analysis results are shown in Figure 11. The results show that, when the mass ratio of Pt/C is 50%, the concentration of phosphoric acid is the highest at the same position, and when the mass ratio of Pt/C is 40%, the air concentration is the lowest. With the increase in the Pt/C mass ratio, the electrochemical surface area gradually increases. Taking into account performance and economic cost factors comprehensively, the optimal design range for Pt/C is 40–50%.

## 5. Conclusions

In this study, an advanced lattice Boltzmann model was proposed to investigate the flow behavior of phosphoric acid in the catalyst layer of HT-PEMFCs. This model is effective for quantitatively predicting the concentration distribution characteristics of phosphoric acid and air, as well as the electrochemical surface area within the catalyst layer, thereby providing theoretical guidance for the structural design of HT-PEMFC catalyst layers.

In this study, the two-dimensional microstructure of CL was established by reconstruction algorithm, and the effects of different carbon carrier diameters, porosity values, and Pt/C mass ratios on the transmission characteristics of phosphoric acid were studied. The main findings are as follows: (1) In this study, the optimum diameter range of carbon carrier was determined to be 50–80 nm, and the phosphoric acid transmission effect was the best in this diameter range, which not only ensured the transmission of phosphoric acid, but also avoided the phenomenon of acid flooding. (2) According to the above analysis of porosity, the optimum porosity range was determined to be 60–70%. The pore structure in this range not only ensured the efficient transmission of phosphoric acid, but also ensured the strength of CL structure. (3) Through the study of Pt/C ratio, the final optimal design range was 40–50%. In this range, the Pt/C structure met the phosphoric acid transmission performance while ensuring efficiency and economy.

This study highlights the importance of balancing the carbon carrier diameter, porosity, and Pt/C mass ratio when designing an efficient and durable HT-PEMFC. By precisely controlling these parameters, the phosphoric acid transport performance in the battery can be effectively improved, and its service life can be extended. In addition, the pore-scale model in this study provides a new perspective for understanding the transport mechanism in HT-PEMFCs. Therefore, this study is of great significance for optimizing the design of CL structure and improving the performance of HT-PEMFCs.

## Figures and Tables

**Table 1:** Reconstruction parameters.

Parameter	Value
$\rho_{C}$	1800
$\rho_{Pt}$	21,450
$\rho_{pol}$	2000
$\theta_{Pt/C}$	0.8
$\theta_{pol}$	0.3

## Data Availability

No new data were created or analyzed in this study. Data sharing is not applicable to this article.

## References

[1] Lee D., Lee G.D. (2016). Carbon composite bipolar plate for high-temperature proton exchange membrane fuel cells (HT-PEMFCs). J. Power Sources.

[2] Wang Y., Seo B., Wang B., Zamel N., Jiao K., Adroher X.C. (2020). Fundamentals, materials, and machine learning of polymer electrolyte membrane fuel cell technology. Energy.

[3] Oh K., Ju H. (2015). Temperature dependence of CO poisoning in high-temperature proton exchange membrane fuel cells with phosphoric acid-doped polybenzimidazole membranes. Int. J. Hydrogen Energy.

[4] Lim K.H., Matanovic I., Maurya S., Kim Y., De Castro E.S., Jang J.-H., Park H., Kim Y.S. (2022). High temperature polymer electrolyte membrane fuel cells with high phosphoric acid retention. ACS Energy Lett..

[5] Brouzgou A., Seretis A., Song S., Shen P.K., Tsiakaras P. (2020). CO tolerance and durability study of PtMe (Me = Ir or Pd) electrocatalysts for H2-PEMFC application. Int. J. Hydrogen Energy.

[6] Aili D., Becker H., Reimer U., Andreasen J.W., Cleemann L.N., Jensen J.O., Pan C., Wang X., Lehnert W., Li Q. (2020). Phosphoric acid dynamics in high temperature polymer electrolyte membranes. J. Electrochem. Soc..

[7] Xue D., Zhang J.N. (2024). Recent progress of antipoisoning catalytic materials for high temperature proton exchange membrane fuel cells doped with phosphoric acid. Ind. Chem. Mater..

[8] Haider R., Wen Y., Ma Z.F., Wilkinson D.P., Zhang L., Yuan X., Song S., Zhang J. (2020). High temperature proton exchange membrane fuel cells: Progress in advanced materials and key technologies. Chem. Soc. Rev..

[9] Li J., Ji J., Li K., Li H., Zhang W., Wang W., Pei Q., Gong C. (2023). Progress in High Temperature Proton Exchange Membranes for Fuel Cell. J. Eng. Sdudies.

[10] Ying J., Liu T., Wang Y., Guo M., Shen Q., Lin Y., Yu J., Yu Z. (2024). Perspectives on Membrane Development for High Temperature Proton Exchange Membrane Fuel Cells. Energy Fuels.

[11] Zakil A.F., Kamarudin S., Basri S. (2016). Modified Nafion membranes for direct alcohol fuel cells: An overview. Renew. Sustain. Energy Rev..

[12] Chevalier S., Fazeli M., Mack F., Galbiati S., Manke I., Bazylak A., Zeis R. (2016). Role of the microporous layer in the redistribution of phosphoric acid in high temperature PEM fuel cell gas diffusion electrodes. Electrochim. Acta.

[13] Søndergaard T., Cleemann L.N., Becker H., Steenberg T., Hjuler H.A., Seerup L., Li Q., Jensen J.O. (2018). Long-Term Durability of PBI-Based HT-PEM Fuel Cells: Effect of Operating Parameters. J. Electrochem. Soc..

[14] Mack F., Klages M., Scholta J., Jörissen L., Morawietz T., Hiesgen R., Kramer D., Zeis R. (2014). Morphology studies on high-temperature polymer electrolyte membrane fuel cell electrodes. J. Power Sources.

[15] Bevilacqua N., George M., Galbiati S., Bazylak A., Zeis R. (2017). Phosphoric Acid Invasion in High Temperature PEM Fuel Cell Gas Diffusion Layers. Electrochim. Acta.

[16] Xia L., Ni M., He Q., Xu Q., Cheng C. (2021). Optimization of gas diffusion layer in high temperature PEMFC with the focuses on thickness and porosity. Appl. Energy.

[17] Xia L., Xu Q., He Q., Ni M., Seng M. (2021). Numerical study of high temperature proton exchange membrane fuel cell (HT-PEMFC) with a focus on rib design. Int. J. Hydrogen Energy.

[18] Sousa T., Mamlouk M., Scott K. (2010). A Non—Isothermal Model of a Laboratory Intermediate Temperature Fuel Cell Using PBI Doped Phosphoric Acid Membranes. Fuel Cells.

[19] Sousa T., Mamlouk M., Scott K. (2009). An isothermal model of a laboratory intermediate temperature fuel cell using PBI doped phosphoric acid membranes. Chem. Eng. Sci..

[20] Sousa T., Mamlouk M., Scott K. (2010). A dynamic non-isothermal model of a laboratory intermediate temperature fuel cell using PBI doped phosphoric acid membranes. Int. J. Hydrogen Energy.

[21] Sousa T., Mamlouk M., Scott K., Rangel C.M. (2012). Three dimensional model of a high temperature PEMFC. Study of the flow field effect on performance. Fuel Cells.

[22] Molaeimanesh G.R., Akbari M.H. (2015). A pore-scale model for the cathode electrode of a proton exchange membrane fuel cell by lattice Boltzmann method. Korean J. Chem. Eng..

[23] Molaeimanesh G.R., Saeidi Googarchin H., Qasemian Moqaddam A. (2016). Lattice Boltzmann simulation of proton exchange membrane fuel cells—A review on opportunities and challenges. Int. J. Hydrogen Energy.

[24] Molaeimanesh G.R., Akbari M.H. (2014). Impact of PTFE distribution on the removal of liquid water from a PEMFC electrode by lattice Boltzmann method. Int. J. Hydrogen Energy.

[25] Wang Y., Qin S., Liao X., Jia Y., Xu H., Wang C., He W., Zhao Y. (2024). Lattice Boltzmann study of the effect of catalyst layer structure on oxygen reduction reaction within a PEMFC. Int. J. Hydrogen Energy.

[26] Yang X., Yang G., Wang H., Xu Z., Ji S., Sun H., Miao H., Yuan J. (2025). Pore-scale investigation of methane steam transport in porous anodes of solid oxide fuel cells with varying structures. J. Power Sources.

[27] Duan K., Zhu L., Li M., Xiao L., Bevilacqua N., Eifert L., Manke I., Markötter H., Zhang R., Zeis R. (2021). Multiphase and Pore Scale Modeling on Catalyst Layer of High-Temperature Polymer Electrolyte Membrane Fuel Cell. J. Electrochem. Soc..

[28] Zhan Z., Song H., Yang X., Jiang P., Chen R., Harandi H.B., Zhang H., Pan M. (2022). Microstructure reconstruction and multiphysics dynamic distribution simulation of the catalyst layer in PEMFC. Membranes.

[29] Salomov U.R., Chiavazzo E., Asinari P. (2014). Pore-scale modeling of fluid flow through gas diffusion and catalyst layers for high temperature proton exchange membrane (HT-PEM) fuel cells. Comput. Math. Appl..

[30] Hou Y., Deng H., Pan F., Chen W., Du Q., Jiao K. (2019). Pore-scale investigation of catalyst layer ingredient and structure effect in proton exchange membrane fuel cell. Appl. Energy.

[31] Kim S.H., Pitsch H. (2009). Reconstruction and effective transport properties of the catalyst layer in PEM fuel cells. J. Electrochem. Soc..

[32] Lange J.K., Sui P., Djilali N. (2010). Pore Scale Simulation of Transport and Electrochemical Reactions in Reconstructed PEMFC Catalyst Layers. J. Electrochem. Soc..

[33] Chen L., Wu G., Holby E.F., Zelenay P., Tao W.-Q., Kang Q. (2015). Lattice Boltzmann pore-scale investigation of coupled physical-electrochemical processes in C/Pt and non-precious metal cathode catalyst layers in proton exchange membrane fuel cells. Electrochim. Acta.

[34] Zhang R., Min T., Chen L., Kang Q., He Y.-L., Tao W.-Q. (2019). Pore-scale and multiscale study of effects of Pt degradation on reactive transport processes in proton exchange membrane fuel cells. Appl. Energy.

[35] Deng H., Hou Y., Chen W., Pan F., Jiao K. (2019). Lattice Boltzmann simulation of oxygen diffusion and electrochemical reaction inside catalyst layer of PEM fuel cells. Int. J. Heat Mass Transf..

[36] Hou Y., Deng H., Du Q., Jiao K. (2018). Multi-component multi-phase lattice Boltzmann modeling of droplet coalescence in flow channel of fuel cell. J. Power Sources.

[37] Hou Y., Deng H., Zamel N., Du Q., Jiao K. (2020). 3D lattice Boltzmann modeling of droplet motion in PEM fuel cell channel with realistic GDL microstructure and fluid properties. Int. J. Hydrogen Energy.

[38] Jiang Z., Yang G., Shen Q., Li S., Liao J., Yang X., Sun J. (2023). Liquid water transport phenomena in the porous transport layer of proton exchange membrane fuel cell based on lattice Boltzmann simulation. Mater. Today Commun..

[39] Liao J., Yang G., Li S., Shen Q., Jiang Z., Wang H., Li Z. (2022). Study of droplet flow characteristics on a wetting gradient surface in a proton exchange membrane fuel cell channel using lattice Boltzmann method. J. Power Sources.

[40] Zhao W., Zhang Y., Xu B., Li P., Wang Z., Jiang S. (2018). Multiple-relaxation-time lattice Boltzmann simulation of flow and heat transfer in porous volumetric solar receivers. J. Energy Resour. Technol..

[41] Liao J., Yang G., Li S., Shen Q., Jiang Z., Wang H. Lattice Boltzmann simulation of droplet growth processes in flow channel of proton exchange membrane fuel cell. Proceedings of the International Conference on Energy Power and Automation Engineering.

[42] Li Q., Luo K.H. (2013). Achieving tunable surface tension in the pseudopotential lattice Boltzmann modeling of multiphase flows. Phys. Rev. E.

[43] Zhao W., Zhang Y., Shang W., Wang Z., Xu B., Jiang S. (2019). Simulation of droplet impacting a square solid obstacle in microchannel with different wettability by using high density ratio pseudopotential multiple-relaxation-time (MRT) lattice Boltzmann method (LBM). Can. J. Phys..

[44] Kharmiani S.F., Passandideh-Fard M., Niazmand H. (2016). Simulation of a single droplet impact onto a thin liquid film using the lattice Boltzmann method. J. Mol. Liq..

